# In vitro antifungal and physicochemical properties of polymerized acrylic resin containing strontium-modified phosphate-based glass

**DOI:** 10.1186/s12903-024-04547-5

**Published:** 2024-07-10

**Authors:** Eun-Jee Jang, Ye-Ji Hong, Yoon-Ha Jeong, Kyoung-Eun Kim, Eun-Seo Jo, Myung-Jin Lee, Song-Yi Yang

**Affiliations:** 1 Department of Dental Hygiene, Konyang University, 158 Gwanjeodong-ro, Seo-gu, Daejeon, 35365 Republic of Korea; 2 Department of Dental Hygiene, Division of Health Science, Baekseok University, Cheonan, 31065 Republic of Korea

**Keywords:** Self-polymerizing acrylic resin, strontium-modified phosphate-based glass, antifungal effect, surface property, physicochemical property

## Abstract

Acrylic resins are widely used as the main components in removable orthodontic appliances. However, poor oral hygiene and maintenance of orthodontic appliances provide a suitable environment for the growth of pathogenic microorganisms. In this study, strontium-modified phosphate-based glass (Sr-PBG) was added to orthodontic acrylic resin at 0% (control), 3.75%, 7.5%, and 15% by weight to evaluate the surface and physicochemical properties of the novel material and its in vitro antifungal effect against *Candida albicans (C. albicans)*. Surface microhardness and contact angle did not vary between the control and 3.75% Sr-PBG groups (*p* > 0.05), and the flexural strength was lower in the experimental groups than in the control group (*p* < 0.05), but no difference was found with Sr-PBG content (*p* > 0.05). All experimental groups showed an antifungal effect at 24 and 48 h compared to that in the control group (*p* < 0.05). This study demonstrated that 3.75% Sr-PBG exhibits antifungal effects against *C. albicans* along with suitable physicochemical properties, which may help to minimize the risk of adverse effects associated with harmful microbial living on removable orthodontic appliances and promote the use of various materials.

## Introduction

The main component of a self-polymerizing resin (a common building material for removable orthodontic appliances) is polymethyl methacrylate (PMMA), which has long been used in dentistry owing to its biocompatibility with oral tissues, stability against discoloration, and suitable mechanical and physical properties [1–3]. However, prolonged intraoral wear, poor maintenance, and drops have resulted in the fracturing of removable orthodontic appliances, which cover large areas of oral mucosal tissue and provide a favorable environment for the colonization and proliferation of pathogenic microorganisms, while plaque, calculus, and microorganisms adhere to the surface of the material, causing oral stomatitis [4–11].

Both bacteria and fungi can cause oral stomatitis. The bacteria include *Gemella haemolysans* and *Streptococcus mitis*, whereas the fungi include *Candida albicans*, *Porphyromonas gingivalis*, *Parvimonas micra*, *Treponema denticola*, *Fusobacterium nucleatum*, and *Candida glabrata* [12, 13]. Treatment of oral stomatitis includes disinfection of appliances, good hygiene habits, and application of topical antifungal agents or systemic medication. However, systemic factors, such as low susceptibility to antibiotics in the elderly, pediatric, and hospitalized patients with long-term admissions, and local factors, such as soft tissue damage, dry mouth, and microbial infections caused by the prosthesis, combine to increase the recurrence rate of stomatitis [14–16].

The use of mouthwashes, such as chlorhexidine, as supplemental antimicrobials is sometimes recommended for people with removable orthodontic appliances. However, chlorhexidine can cause side effects, such as altered taste and tooth discoloration [17]. Therefore, a need exists for antibacterial substances that improve the antimicrobial properties of the orthodontic appliance without adversely affecting its mechanical properties. Previous studies proposed the addition of antimicrobial materials, such as chitosan, silver nitrate, silver nanoparticles, zinc oxide, and titanium dioxide nanoparticles to the resins of removable orthodontic appliances [17–19]. These studies showed excellent antibacterial and antifungal properties; however, some reported low biocompatibility and a tendency to reduce material strength, which promotes fracturing. Therefore, when adding antimicrobials, careful consideration should be given to how they affect the durability of the structure [20].

Phosphate-based glass (PBG) is suitable for use in biomaterial and tissue engineering because its chemical composition is similar to that of natural bone and teeth, and its calcium and phosphate content promotes the remineralization of teeth. In particular, phosphate contributes substantially to the formation of hydroxyapatite and increases biocompatibility [21]. However, the application of pure PBG is limited by its low chemical durability. The addition of strontium (Sr) to PBGs has been reported to improve chemical durability [22]. Sr can also promote remineralization of dentin and enamel and has been shown to stimulate dentin production by human dental pulp stem cells [23]. Antimicrobial activity is greatest in cements containing strontium, especially against specific species [24], and Sr-replacement bioactive glasses have been shown to inhibit the growth of *Aggregatibacter actinomycetemcomitans* and *P. gingivalis*, depending on the strontium ratio [25]. The addition of Sr-PBG to dental composite resin also enhanced long-term antibacterial activity and inhibited the occurrence of secondary caries, confirming its potential in clinical practice [26–28].

To date, various antimicrobial substances have been added to dental materials to study their antimicrobial and physicochemical properties. However, no study has investigated the addition of Sr-PBG to self-polymerizing acrylic resins. The aim of this study was to systematically evaluate the antimicrobial effectiveness of Sr-PBG incorporated at various concentrations in a self-polymerizing acrylic resin against *C. albicans*, while also assessing its impact on the surface, physicochemical, and mechanical properties of the material.

## Methods

### Glass preparation

To prepare the Sr-PBG powder, phosphorus pentoxide (P_2_O_5_; 50 mol%), calcium oxide (CaO; 15 mol%), sodium oxide (Na_2_O; 20 mol%), and strontium oxide (SrO; 15 mol%) powders (Sigma-Aldrich, St. Louis, MO, USA) were mixed in a high-speed mixer (Hauschild, Hamm, Germany) for 5 min. The mixed batches were melted in an alumina crucible at 1,100 °C for 1 h, and the melted glass was quenched at room temperature to obtain a glass cullet. The obtained glass cullets were ground in an alumina mortar and pulverized under dry conditions using a planetary mono mill (Pulverisette 7; FRITSCH, Idar-Oberstein, Germany).

The size and morphology of the obtained Sr-PBG powder were determined using a particle size analyzer (Mastersizer 2000, Malvern Instruments, Malvern, UK) and field-emission scanning electron microscope (FE-SEM; JSM-7800 F; JEOL, Tokyo, Japan) with energy-dispersive X-ray spectroscopy (EDS) at 500× magnification and an accelerating voltage of 10.0 kV.

### Specimen preparation

As the self-polymerized acrylic resin, Ortho-Jet™ Package (Lang Dental Manufacturing, Wheeling, IL, USA) was selected for corrective use. To produce an acrylic resin containing Sr-PBG, 0% (control), 3.75%, 7.5%, and 15% Sr-PBG powders were mixed in different weight ratios (Table 1). The acrylic resin powder and Sr-PBG powder were mixed with acrylic resin liquid at a 2 (liquid):3 (powder) ratio. The polymerization was performed at 60 °C with an air press unit (4.0 bar, 15 min; Air press unit, Sejong Dental, Iksan-si, Korea). The polymerized sample was removed from each experimental mold, and the front, back, and sides were polished so that the surface was uniform in the order of #800, #1,000, #1,200, and #1,500 SiC paper using a polishing machine (EcoMet Manual Single Grinder Polisher, Buehler, Lake Bluff, IL, USA).

**Table 1:** Composition of the control and experimental groups

Group	Sr-PBG powder (wt.%)	Self-polymerized acrylic resin powder (wt.%)	Self-polymerized acrylic resin liquid (wt.%)
control	0	60.0	40.0
Sr-PBG 3.75%	3.75	56.25	40.0
Sr-PBG 7.50%	7.50	52.50	40.0
Sr-PBG 15%	15.0	45.0	40.0

#### Contact angle

To measure the contact angle, a disc-shaped specimen (diameter: 1 cm, thickness: 1 mm) was manufactured and placed in a contact-angle analyzer (SmartDrop, Femtobiomed, Gyonggi-do, Korea). Distilled water (5 µL) was randomly dropped onto the surface of each specimen and measured after 10 s of contact. This process was repeated twice for each specimen, and the average was selected as the representative value (*n* = 10).

#### Surface microhardness

To measure surface microhardness, a disc-shaped specimen (diameter: 1 cm, thickness: 1 mm) was placed in a hardness tester (Mmt-x7B, Matsuzawa, Tokyo, Japan). The microhardness of the surface was measured using a Vickers hardness tester with a diamond pyramid and vertex-to-surface angle of 136°. To measure the Vickers hardness of the specimen, the indentation load of the hardness tester was set to 50 g, and the indentation time was set to 10 s. Each specimen was then randomly measured twice, and the average was selected as the representative value (*n* = 10).

### Flexural strength

To measure three-point flexural strength, a specimen (height: 3.3 mm, width: 10 mm, length: 64 mm) was ground with SiC #1,500 grit paper according to ISO 20795-1:2013 (Dentistry - Base polymers - Part 1: Denture base polymers), and then entirely immersed in distilled water at 37 ± 1 °C. After 24 h, the specimen was removed from the distilled water and loaded into a universal testing machine (Model 5942, Instron, Norwood, MA, USA) to measure flexural strength (*n* = 10). The span of the universal testing machine was set to 50 mm, and the crosshead speed was set to 5 mm/min. The load was measured in N, and the flexural strength was calculated using the following formula:

$$Flexural{\text{ }}strength{\text{ }}\left( {MPa} \right){\text{ }} = {\text{ }}3Fl/2b{h^2}$$

where *F* is the maximum load (N), *l* is the span length (50 mm), *b* is the width (10 mm), and *h* is the height of the specimen (3.3 mm).

### SEM-EDS observation

The surface of a disc-shaped specimen (diameter: 1 cm, thickness: 1 mm) was inspected using the SEM-EDS device. The micromorphology and chemical components of the experimental specimen surfaces were observed at a magnification of 500× under an acceleration voltage of 10.0 kV.

### Evaluation of antimicrobial effect

*C. albicans* (ATCC 10,231) were cultured in YM culture medium (BD, Difco, Franklin Lakes, NJ, USA) at 37 ± 1 °C for more than 48 h. After culturing, optical density (OD_600_) was measured using a spectrophotometer (BioMate 3 SUV-Vis, Thermo Fisher Scientific, Madison, WI, USA). After assessing sample absorbance in the range of 0.4 to 0.6, a sterilized disc-shaped sample (diameter: 10 mm, thickness: 1 mm) was completely immersed in 1 mL of *C. albicans* floating solution and incubated at 37 ± 1 °C for 24 h and 48 h (*n* = 6). After each culture period, 100 µL of *C. albicans* floating solution was spread on YM agar plates (BD, Difco) and stored at 37 ± 1 °C for 24 h. The *C. albicans* colony-forming units (CFU) on the YM agar plates were counted and compared.

### Evaluation of ion release

A disc-shaped specimen (diameter: 10 mm, thickness: 1 mm) was entirely immersed in 5 mL of distilled water at 37 ± 1 °C. After 24 h, the specimens were removed and the amounts of calcium (Ca), phosphorus (P), and Sr released into distilled water were detected using an inductively coupled plasma optical emission spectrometer (ICP-OES; Optima 8300; PerkinElmer, Waltham, MA, USA) (*n* = 6).

### Statistical analysis

All statistical analyses were performed using IBM SPSS Statistics for Windows (version 25.0; IBM SPSS, Armonk, NY, USA). For all groups, differences were analyzed using one-way analysis of variance (ANOVA) followed by Tukey’s post-hoc test, with statistical significance set at *p* < 0.05.

## Results

### Sr-PBG powder characterization

**Fig. 1:**
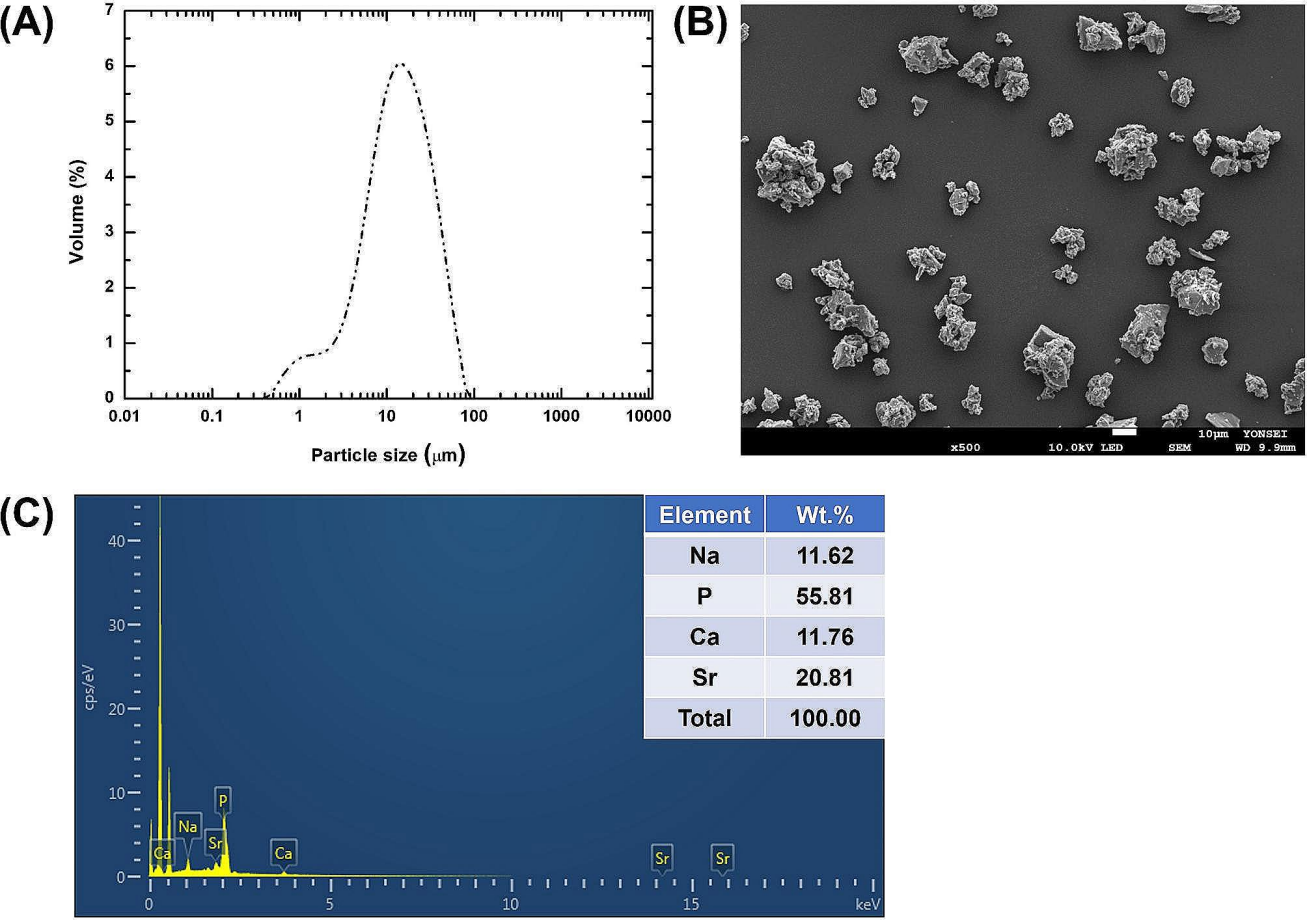
Characterization of Sr-PBG powder. **(A)** Particle size distribution, **(B)** Scanning electron microscopy (SEM) image. **(C)** Energy-dispersive X-ray spectroscopy (EDS) image

Figure 1(A) presents the particle size distribution of the Sr-PBG powders, which exhibited a range of 2.09 to 52.16 μm with a median diameter (d50) of 14.70 μm. Figure 1(B) shows the morphology of the Sr-PBG powders. SEM showed irregularly shaped and aggregated particles, with a size of approximately 10 μm. Na, P, Ca, and Sr were observed in the Sr-PBG (Fig. 1C).

### Contact angle

**Fig. 2:**
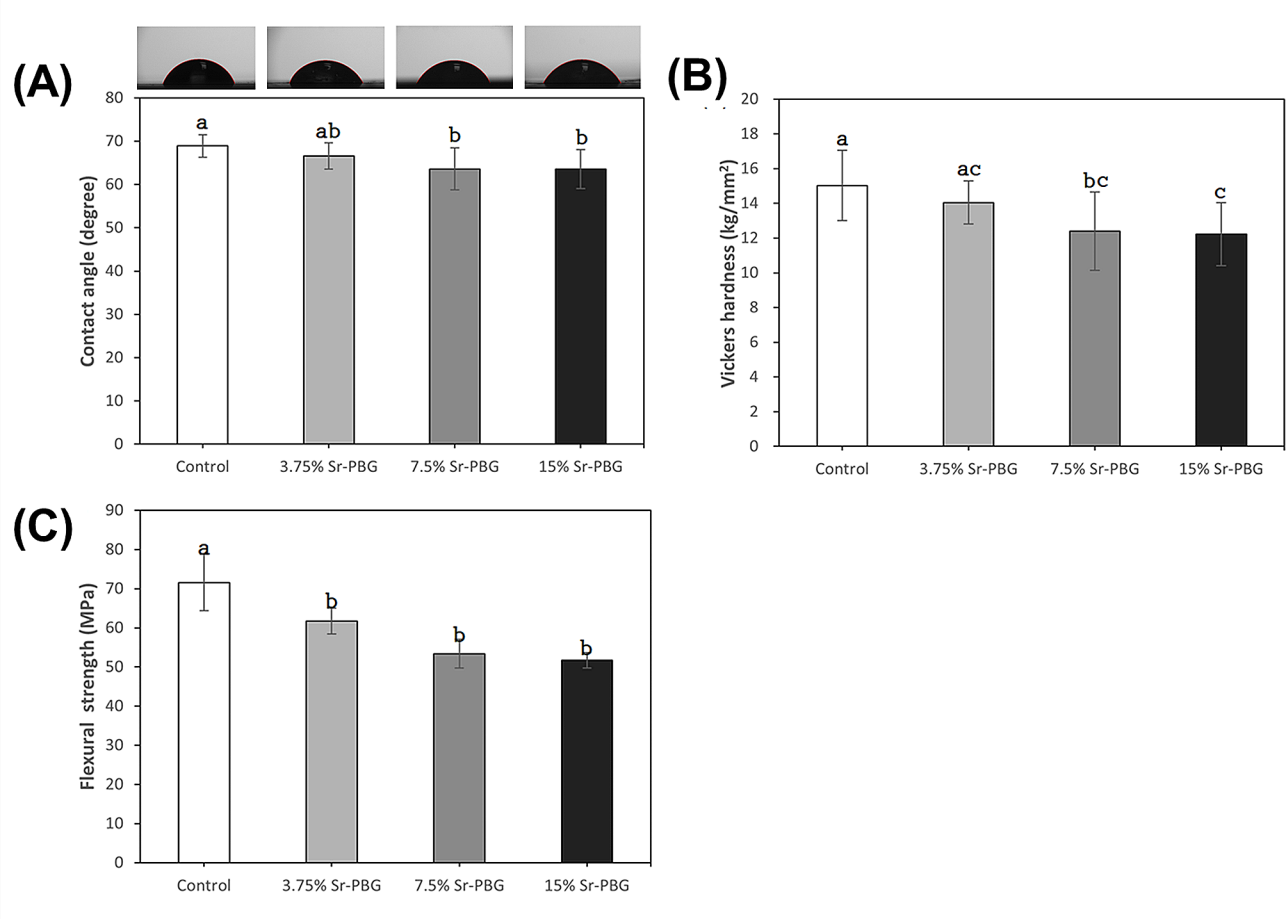
Surface, physical, and mechanical properties of acrylic resin specimens with increasing Sr-PBG content. **(A)** Contact angle, **(B)** Vickers hardness, and **(C)** three-point flexural strength. Data are presented as the mean ± standard deviation (SD; *n* = 10). Different lowercase letters indicate significant differences at *p* < 0.05

Figure 2(A) presents the contact angles of the specimen surfaces, which were 68.88 ± 2.63° for the control group, 66.56 ± 3.04° for the 3.75% Sr-PBG, 63.56 ± 4.83° for the 7.5% Sr-PBG, and 63.52 ± 4.52° for the 15% Sr-PBG. There was no significant difference between the control and 3.75% Sr-PBG groups (*p* > 0.05); 7.5% Sr-PBG and 15% Sr-PBG showed significantly lower contact angles than those of the control group (*p* < 0.05). The contact angles of the 3.75% Sr-PBG, 7.5% Sr-PBG, and 15% Sr-PBG did not vary significantly with concentration (*p* > 0.05), despite the increasing content of Sr-PBG.

### Vickers hardness

Figure 2(B) presents the Vickers hardness of specimen surfaces. The control group showed the highest hardness (15.02 ± 2.02), followed by 3.75% (14.04 ± 1.24), 7.5% (12.39 ± 2.26), and 15% Sr-PBG (12.22 ± 1.18), indicating a decrease in hardness with increasing Sr-PBG content. The 7.5% and 15% Sr-PBG had lower hardness values than those of the control (*p* < 0.05); the microhardness value decreased with increasing Sr-PBG concentration (*p* < 0.05). In contrast, the hardness values of the control and 3.75% Sr-PBG did not vary (*p* > 0.05).

### Flexural strength

Figure 2(C) presents the three-point flexural strength of the specimens, which was 71.55 ± 7.23 MPa for the control group, 61.72 ± 3.26 MPa for the 3.75% Sr-PBG, 53.37 ± 3.68 MPa for the 7.5% Sr-PBG, and 51.71 ± 2.00 MPa for the 15% Sr-PBG. The 3.75% Sr-PBG, 7.5% Sr-PBG, and 15% Sr-PBG showed lower flexural strength than that of the control group (*p* < 0.05), but did not vary with increasing Sr-PBG content (*p* > 0.05).

### SEM-EDS observation

**Fig. 3:**
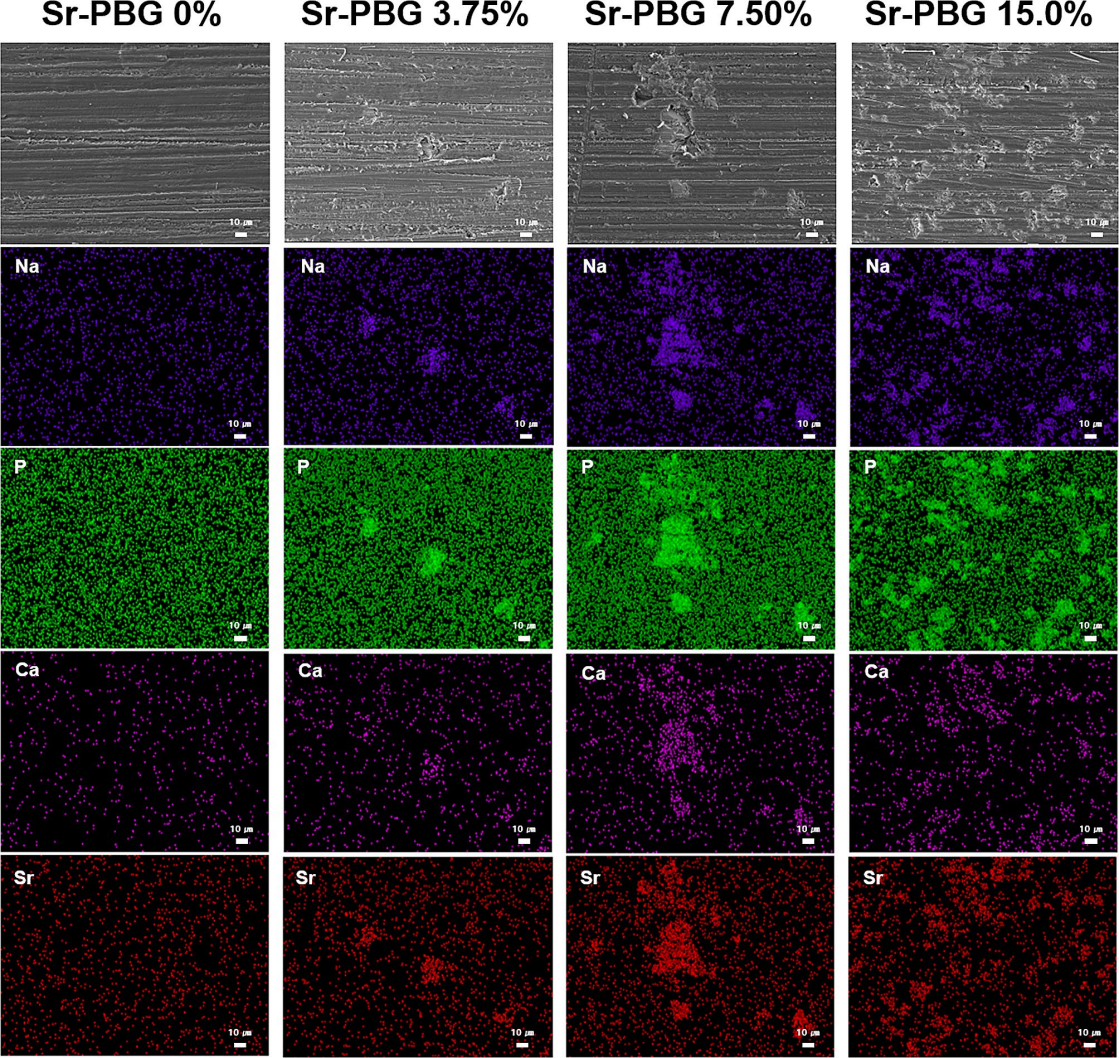
SEM-EDS mapping images of the control and experimental specimen surfaces. Top: SEM image; purple indicates Na, green indicates P, pink indicates Ca, and red indicates Sr

Figure 3 presents the SEM-EDS image of a specimen surface, which showed signs of polishing in both the control and experimental groups. As the Sr-PBG content increased, the aggregated Sr-PBG became embedded in the acrylic resin surface. Similarly, the EDS mapping images showed that the components comprising Sr-PBG were embedded on the surface, and a denser distribution of Na, P, Ca, and Sr was observed with increasing Sr-PBG content.

### Evaluation of antifungal effect

**Fig. 4:**
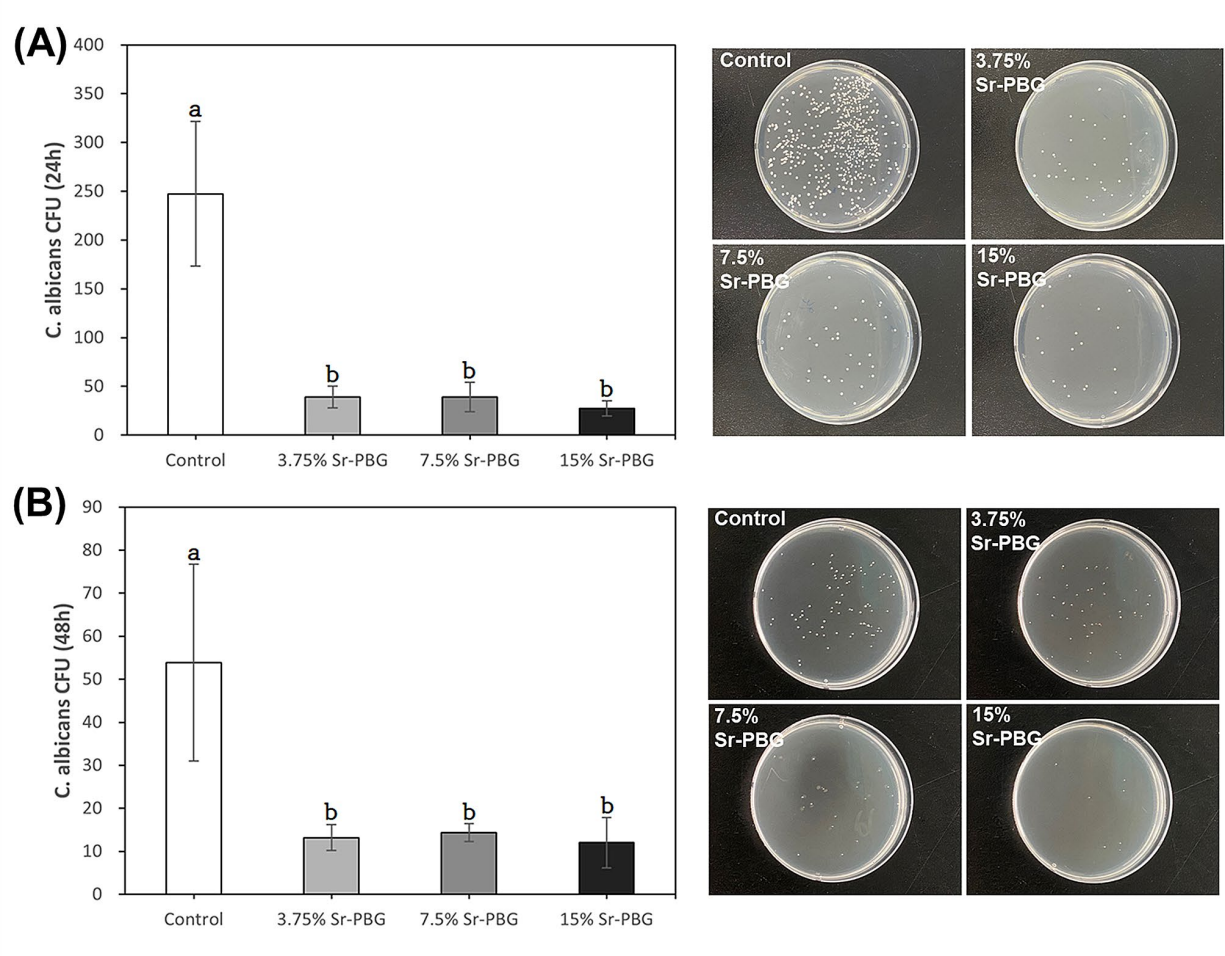
CFU counts and images of control and experimental specimens after culturing with *Candida albicans* for **(A)** 24 h and **(B)** 48 h. Data are presented as the mean ± SD (*n* = 6). Different lowercase letters indicate significant differences at *p* < 0.05

After incubation of *C. albicans* with the control and Sr-PBG-containing specimens for 24 h, CFUs were determined to be 247.3 ± 74.2 for the control group, 39.0 ± 11.2 for the 3.75% Sr-PBG, 39.0 ± 15.0 for the 7.5% Sr-PBG, and 27.5 ± 7.7 for the 15% Sr-PBG, indicating that the control group had the highest CFU value (*p* < 0.05; Fig. 4A). There was no significant difference in the CFU values among the specimens containing different Sr-PBG concentrations (*p* > 0.05).

After incubation of *C. albicans* with control and Sr-PBG-containing specimens for 48 h, CFUs were measured at 53.8 ± 22.9 for the control group, 13.2 ± 3.0 for the 3.75% Sr-PBG, 14.3 ± 2.1 for the 7.5% Sr-PBG, and 12.0 ± 5.9 for the 15% Sr-PBG (Fig. 4B). The control group had the highest CFU value among the groups (*p* < 0.05). There was no difference in the CFU values with increasing Sr-PBG concentrations (*p* > 0.05).

### Ion release

**Fig. 5:**
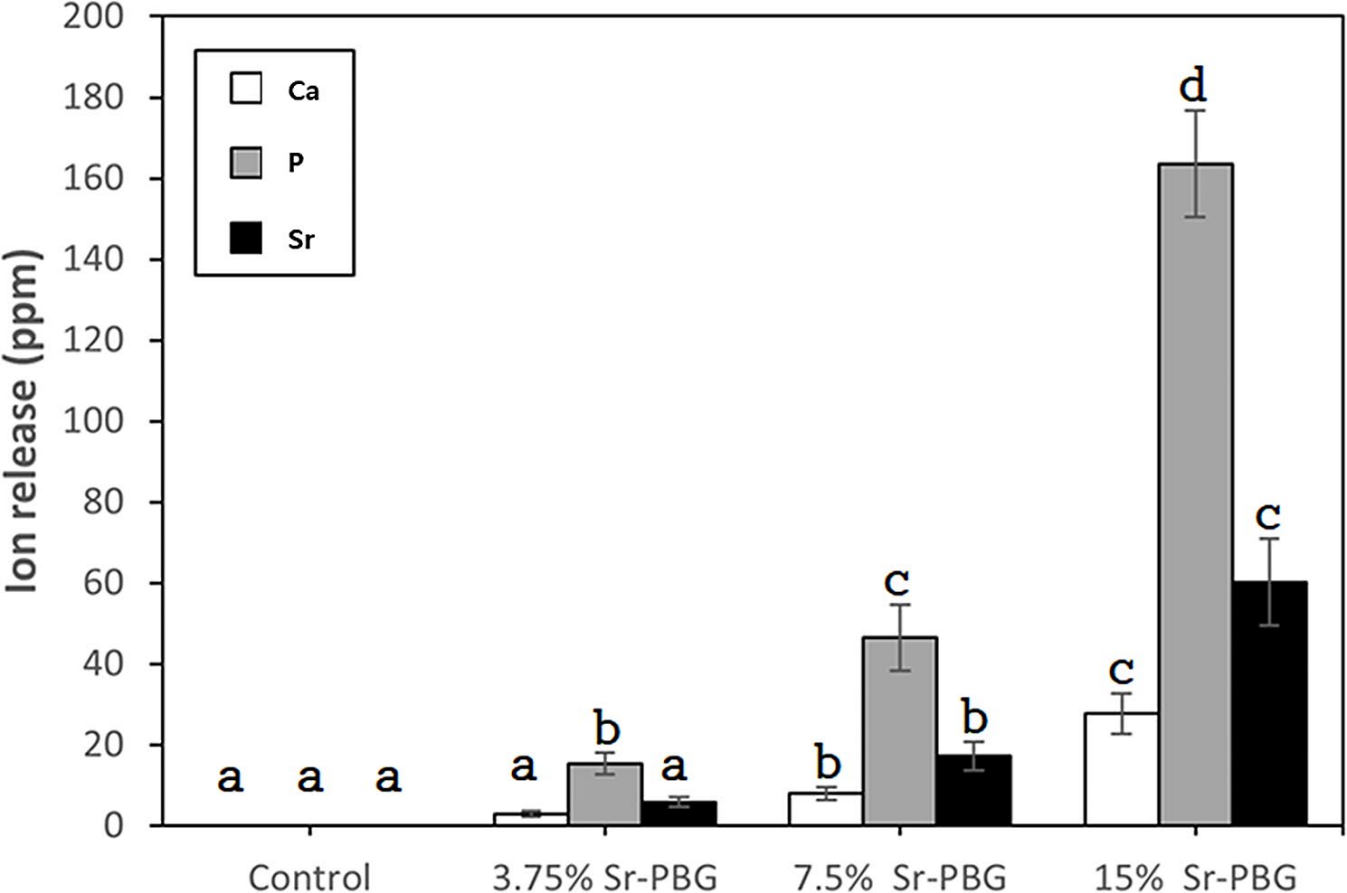
Ca, P, and Sr ion release from control and experimental specimens after immersion in distilled water. Data are presented as the mean ± SD (*n* = 6). Different lowercase letters indicate a significant difference in the amount of each ion released between the control and experimental groups (*p* < 0.05)

Figure 5 shows the number of ions released by the control and experimental groups. The ion release measurements for Ca were 2.96 ± 0.71 ppm for the 3.75% Sr-PBG, 8.00 ± 1.59 ppm for the 7.5% Sr-PBG, and 27.72 ± 5.01 ppm 15% Sr-PBG. The 3.75% Sr-PBG showed no significant difference compared to the control group (*p* > 0.05), while the 7.5% and 15% Sr-PBG showed significantly higher Ca ion release values (*p* < 0.05), and the amount of Ca released increased significantly with increasing Sr-PBG concentration (*p* < 0.05).

For P, 3.75% Sr-PBG released 15.38 ± 2.73 ppm, 7.5% Sr-PBG released 46.43 ± 8.11 ppm, and 15% Sr-PBG released 163.62 ± 13.20 ppm. The amount of P released increased significantly with increasing concentrations of Sr-PBG, compared with that in the control group (*p* < 0.05).

For Sr, 3.75% Sr-PBG released 5.82 ± 1.27 ppm, 7.5% Sr-PBG released 17.18 ± 3.54 ppm, and 15% Sr-PBG released 60.13 ± 10.73 ppm. The 3.75% Sr-PBG showed no significant difference from the control group (*p* > 0.05), while P ion release increased with increasing Sr-PBG concentrations (*p* < 0.05).

## Discussion

Orthodontic treatment using removable orthodontic retainers is performed to improve the alignment of the teeth and jaw. However, the use of removable orthodontic retainers can alter the conditions of the patient’s mouth and promote stomatitis. Stomatitis is a major complication in orthodontic treatment, which can lead to patient discomfort and increased medical costs [29–31]. The prolonged use of removable retainers also creates a suitable environment for *C. albicans* colonization and proliferation [32]. Therefore, this study aimed to develop a more effective antimicrobial-loaded material for removable orthodontic appliances to prevent and treat *C. albicans* infections while providing suitable physicochemical properties for clinical use. In this study, we evaluated different concentrations of Sr-PBG to find the clinically acceptable range between antimicrobial efficacy and material properties. This approach helped determine the concentration necessary to maintain clinical efficacy without compromising material properties. Investigating both higher and lower concentrations was critical in determining the most effective range for dental applications.

We evaluated the surface, physicochemical, and antimicrobial properties of self-polymerizing orthodontic acrylic resins prepared with different concentrations of Sr-PBG. Contact angle reflects the energy of a solid surface and is used to determine the hydrophilicity and hydrophobicity of a material. Low wettability of removable orthodontic appliances significantly affects their retention in the oral cavity, increasing friction against the oral mucosa, particularly under reduced salivary flow [33]. This friction, intensified at higher contact angles, diminishes mucosal lubrication and exacerbates mucosal trauma, thus reducing appliance comfort and effectiveness and increasing the risk of mucosal injury. The 7.5% and 15% Sr-PBG showed significantly lower contact angles than those in the control group, suggesting that Sr-PBG increases the hydrophilicity of self-polymerizing orthodontic acrylic resins, thereby promoting wettability along with the retention of removable orthodontic appliances and reduces the potential for frictional damage to the oral mucosa.

Wear is a complex phenomenon that occurs naturally owing to substances in the oral cavity and is closely related to the surface microhardness of the material [34]. Surface microhardness is a common indicator of a material’s durability and is determined by observing physical changes under various procedures, such as pressure-induced denting, scratching, abrasion, and penetration. The constant pressure applied in the oral cavity affects acrylic resins and can impact the abrasiveness of the material [35]. In this study, no significant differences in surface hardness were found between the control group and 3.75% Sr-PBG. However, 7.5% and 15% Sr-PBG showed significantly lower surface microhardness values than those in the control group. Furthermore, 3.75% Sr-PBG, 7.5% Sr-PBG, and 15% Sr-PBG showed no significant differences in surface microhardness. These results indicate that Sr-PBG can affect the surface microhardness of PMMA acrylic resins; therefore, determining the optimal Sr-PBG content could potentially improve the durability of acrylic resin and extend the service life of dental materials.

It is important to determine the flexural strength of acrylic resins with antimicrobial additives because it affects the potential for fracture in removable orthodontic retainers. Previous studies reported that the addition of Sr to PBG improved its chemical durability; however, the effects on physical durability are unclear [22]. In this study, there was no significant difference in the flexural strength of 3.75% Sr-PBG compared to that of the control group; however, 7.5% and 15% Sr-PBG showed significantly lower flexural strength values. These results indicate that flexural strength tends to decrease with increasing Sr-PBG concentration. Higher concentrations of Sr-PBG in acrylic resin reduce flexural strength due to insufficient interfacial bonding, leading to the formation of micro-gaps that act as stress concentrators, thereby increasing the potential for fracture [6]. Despite this challenge, in our study, the flexural strength of the experimental group containing 3.75% Sr-PBG was measured to be 61.72 ± 3.26 MPa, surpassing the minimum requirement of 60 MPa specified by ‘ISO 20795-1’ for ‘Type 2: Autopolymerizable materials’. This indicates that even though higher concentrations of Sr-PBG may weaken interfacial bonding, the 3.75% concentration maintains adequate flexural strength for clinical use.

PMMA is widely used as a material for removable orthodontic appliances; however, its surface properties can alter the oral environment, facilitating *C. albicans* colonization and proliferation. This microbial activity is significantly associated with the development of stomatitis, especially under conditions that reduce saliva flow, thereby increasing the risk of oral infections [7]. PBG alone cannot solve this problem because of its low chemical durability and limited thermal stability. However, adding Sr can greatly improve chemical durability and thermal stability, among other physical properties, and has weak antibacterial activity [23]. The antimicrobial effect of Sr has been observed against a wide range of microorganisms, and its inhibitory effect on oral microorganisms is well-recognized [36]. After incubating the control and Sr-PBG-containing specimens with *C. albicans* for 24 and 48 h, we measured the CFUs and found that the Sr-PBG-containing experimental groups showed significantly lower values than the control, with no significant difference in CFU values between the different Sr-PBG concentrations. We inferred that the Sr released from the Sr-PBG contributed to the growth inhibition of *C. albicans*. Sr ions can penetrate the cells of microorganisms and interfere with important biological processes, such as nucleic acid synthesis, protein production, and enzyme activity. Sr ions can also interfere with important ion exchange processes, thereby limiting cellular functions and inhibiting the survival and reproduction of affected microorganisms.

Ion release is important for assessing the biocompatibility and oral health risks of orthodontic devices because it affects the chemical and physical properties of the material. In the oral cavity, ions can potentially interact with oral tissues and cells to cause adverse effects. Therefore, ion release should be analyzed in various environments to assess the suitability of orthodontic devices [37]. In this study, the 3.75% Sr-PBG group was not significantly different from the control group in terms of Ca-ion release, but the 7.5% and 15% Sr-PBG groups showed significantly higher Ca-ion release rates. P ion release significantly increased in all experimental groups compared to that in the control. Sr ion release was not significantly different from that of the control, but significantly higher ion release rates were observed for 3.75% Sr-PBG, 7.5% Sr-PBG, and 15% Sr-PBG. In addition, the number of *C. albicans* tended to decrease as Sr-PBG content increased. Thus, PMMA containing Sr-PBG exhibited antifungal effects against *C. albicans* [38].

This study evaluated the in vitro antimicrobial, surface, and physicochemical properties of a newly developed material for removable orthodontic retainers. Despite the demonstrated biocompatibility and safety of similar materials in previous studies [39], the long-term effects and stability of strontium-modified phosphate-based glass in orthodontic applications are less well understood. A major limitation of our in vitro approach is the exclusion of natural factors such as saliva, which could potentially alter the properties of the material and its antimicrobial efficacy. The present study does not address how the interaction with saliva might affect the stability and antimicrobial properties of Sr-PBG added to orthodontic acrylic resin over extended periods of time. In addition, potential adverse effects, such as changes in the properties of the material over time and its interaction with the oral environment, require further investigation. Therefore, considering these factors, further studies are required to better understand the long-term stability, antimicrobial efficacy, and potential clinical benefits of incorporating Sr-PBG into orthodontic acrylic resins in the presence of saliva and other biological factors. Nevertheless, based on our results, we propose that low-concentration Sr-PBG-containing materials will improve the performance and efficacy of removable orthodontic retainers in clinical applications, ultimately minimizing oral health risks and increasing patient satisfaction.

## Conclusion

The aim of this study was to systematically evaluate the in vitro antimicrobial effectiveness of Sr-PBG incorporated at various concentrations in a self-polymerizing acrylic resin against C. albicans, while also assessing its impact on the surface, physicochemical, and mechanical properties of the material. The results demonstrated significant antifungal effects of Sr-PBG in the self-polymerizing orthodontic acrylic resin, coupled with the maintenance of surface hardness and flexural strength suitable for clinical application. Consequently, this study suggests that self-polymerizing orthodontic acrylic resins containing Sr-PBG offer a promising solution for orthodontic treatment and oral healthcare.

## Data Availability

The data will be made available by the corresponding author upon reasonable request.

## References

[1] Zachrisson BU (2005). Global trends and paradigm shifts in clinical orthodontics. World J Orthod.

[2] Lino MM, Paulo CS, Vale AC, Vaz MF, Ferreira LS (2013). Antifungal activity of dental resins containing amphotericin B-conjugated nanoparticles. Dent Mater.

[3] Raszewski Z, Nowakowska-Toporowska A, Nowakowska D, Więckiewicz W (2021). Update on acrylic resins used in dentistry. Mini Rev Med Chem.

[4] Peutzfeldt A (1997). Resin composites in dentistry: the monomer systems. Eur J Oral Sci.

[5] Iça RB, Öztürk F, Ates B, Malkoc MA, Kelestemur Ü (2014). Level of residual monomer released from orthodontic acrylic materials. Angle Orthod.

[6] de Castro DT, Valente ML, Agnelli JAM, da Silva CHL, Watanabe E, Siqueira RL, Reis D (2016). A. C. In vitro study of the antibacterial properties and impact strength of dental acrylic resins modified with a nanomaterial. J Prosthet Dent.

[7] Hibino K, Wong RW, Haegg U, Samaranayake LP (2009). The effects of orthodontic appliances on Candida in the human mouth. Int J Paediatr Dent.

[8] Gong SQ, Epasinghe J, Rueggeberg FA, Niu LN, Mettenberg D, Yiu CK, Tay FR (2012). An ORMOSIL-containing orthodontic acrylic resin with concomitant improvements in antimicrobial and fracture toughness properties. PLoS One.

[9] Tsomos G, Ludwig B, Grossen J, Pazera P, Gkantidis N (2014). Objective assessment of patient compliance with removable orthodontic appliances: a cross-sectional cohort study. Angle Orthod.

[10] Webb BC, Thomas CJ, Willcox MDP, Harty DWS, Knox KW (1998). Candida-associated denture stomatitis. Aetiology and management: a review. Part 1. Factors influencing distribution of Candida species in the oral cavity. Aust Dent J.

[11] Farronato G, Giannini L, Galbiati G, Cannalire p, Martinelli G, Tubertini I, Maspero C (2013). Oral tissues and orthodontic treatment: common side effects. Minerva Stomatal.

[12] Napeñas JJ, Brennan MT, Coleman S, Kent ML, Noll J, Frenette G, Bahrani-Mougeot FK (2010). Molecular methodology to assess the impact of cancer chemotherapy on the oral bacterial flora: a pilot study. Oral Surg Oral Med Oral Pathol Oral Radiol Endod.

[13] Laheij AM, de Soet JJ, von dem Borne PA, Kuijper EJ, Kraneveld EA, van Loveren C, Raber-Durlacher JE (2012). Oral bacteria and yeasts in relationship to oral ulcerations in hematopoietic stem cell transplant recipients. Support Care Cancer.

[14] Brown MRW, Gilbert P (1993). Sensitivity of biofilms to antimicrobial agents. J Appl Bacteriol.

[15] Hawser SP, Douglas LJ (1995). Resistance of Candida albicans biofilms to antifungal agents in vitro. Antimicrob Agents Chemother.

[16] Al-Dwairi ZN, Al-Quran FA, Al-Omari OY (2012). The effect of antifungal agents on surface properties of poly (methyl methacrylate) and its relation to adherence of Candida albicans. J Prosthodont Res.

[17] Esmaeilzadeh M, Divband B, Ranjkesh B, Pournaghi Azar F, Yeganeh Sefidan F, Kachoei M, Karimzadeh B (2022). Antimicrobial and mechanical properties of orthodontic acrylic resin containing zinc oxide and titanium dioxide nanoparticles supported on 4A zeolite. Int J Dent.

[18] Walczak K, Schierz G, Basche S, Petto C, Boening K, Wieckiewicz M (2020). Antifungal and surface properties of chitosan-salts modified PMMA denture base material. Molecules.

[19] Monteiro DR, Gorup LF, Takamiya AS, de Camargo ER, Filho ACR, Barbosa DB (2012). Silver distribution and release from an antimicrobial denture base resin containing silver colloidal nanoparticles. J Prosthodont.

[20] Butler J, Handy RD, Upton M, Besinis A (2023). Review of antimicrobial nanocoatings in medicine and dentistry: mechanisms of action, biocompatibility performance, safety, and benefits compared to antibiotics. ACS Nano.

[21] Lee MJ, Seo YB, Seo JY, Ryu JH, Ahn HJ, Kim KM, Choi SH (2020). Development of a bioactive flowable resin composite containing a zinc-doped phosphate-based glass. Nanomaterials.

[22] El Jouad M, Touhtouh S, Hajjaji A (2023). First investigation of the effect of strontium oxide on the structure of phosphate glasses using molecular dynamics simulations. Comput Mater Sci.

[23] Krishnan V, Bhatia A, Varma H (2016). Development, characterization and comparison of two strontium doped nano hydroxyapatite molecules for enamel repair/regeneration. Dent Mater.

[24] Dabsie F, Gregoire G, Sixou M, Sharrock P (2009). Does strontium play a role in the cariostatic activity of glass ionomer? Strontium diffusion and antibacterial activity. J Dent.

[25] Liu J, Rawlinson SC, Hill RG, Fortune F (2016). Strontium-substituted bioactive glasses in vitro osteogenic and antibacterial effects. Dent Mater.

[26] Tavoni M, Dapporto M, Tampieri A, Sprio S (2021). Bioactive calcium phosphate-based composites for bone regeneration. J Compos Sci.

[27] Arrais CAG, Micheloni CD, Giannini M, Chan DC (2003). Occluding effect of dentifrices on dentinal tubules. J Dent.

[28] Ammann P (2005). Strontium ranelate: a novel mode of action leading to renewed bone quality. Osteoporos Int.

[29] Krey KF, Hirsch C (2012). Frequency of orthodontic treatment in German children and adolescents: influence of age, gender, and socio-economic status. Eur J Orthod.

[30] Chestnutt IG, Burden DJ, Steele JG, Pitts NB, Nuttall NM, Morris AJ (2003). The orthodontic condition of children in the United Kingdom. Br Dent J.

[31] Bajunaid SO, Baras BH, Weir MD, Xu HH (2022). Denture acrylic resin material with antibacterial and protein-repelling properties for the prevention of denture stomatitis. Polymers.

[32] Yang SY, Choi JW, Kim KM, Kwon JS (2022). Evaluation of the time-dependent efficacy of commercial denture or orthodontic appliance cleansers: an in vitro study. Dent Mater J.

[33] Shinonaga Y, Arita K (2012). Antibacterial effect of acrylic dental devices after surface modification by fluorine and silver dual-ion implantation. Acta Biomater.

[34] Dayan C, Kiseri B, Gencel B, Kurt H (2019). Tuncer, N. Wear resistance and microhardness of various interim fixed prosthesis materials. J Oral Sci.

[35] Lai C, Nguyen A, Ye L, Hao J, Koo H, Mante F, Ozer F (2022). Antibacterial and physical properties of PVM/MA copolymer-incorporated polymethyl methacrylate as a novel antimicrobial acrylic resin material. Molecules.

[36] Alshammari H, Bakitian F, Neilands J, Andersen OZ, Stavropoulos A (2021). Antimicrobial properties of strontium functionalized titanium surfaces for oral applications, a systematic review. Coatings.

[37] Staffolani N, Damiani F, Lilli C, Guerra M, Staffolani NJ, Belcastro S, Locci P (1999). Ion release from orthodontic appliances. J Dent.

[38] Lee MJ, Kim MJ, Mangal U, Seo JY, Kwon JS, Choi SH (2022). Zinc-modified phosphate-based glass micro-filler improves Candida albicans resistance of auto-polymerized acrylic resin without altering mechanical performance. Sci Rep.

[39] Go HB, Lee MJ, Seo JY, Byun SY, Kwon JS (2023). Mechanical properties and sustainable bacterial resistance effect of strontium-modified phosphate-based glass microfiller in dental composite resins. Sci Rep.

